# P025: Norovirus inactivation on antimicrobial touch surfaces

**DOI:** 10.1186/2047-2994-2-S1-P25

**Published:** 2013-06-20

**Authors:** B Keevil, S Warnes

**Affiliations:** 1 Centre for Biological Sciences, University of Southampton, Southampton, UK

## Introduction

Norovirus is the most common cause of gastroenteritis worldwide, primarily because of high infectivity, uncontrollable aerosol formation via vomitus and faeces, resistance to cleaning agents and persistence in the environment. Even low level surface contamination is a transmission risk because of the low infectious dose and inadequate hand hygiene.

## Objectives

Laboratory studies and clinical trials have demonstrated the use of antimicrobial copper alloy touch surfaces to reduce the spread of bacterial pathogens and antibiotic resistance gene transfer. Here we investigate the efficacy of copper alloys to inactivate norovirus.

## Methods

In the absence of infectivity assays for human norovirus, Infectivity of surrogate murine MNV-1 norovirus, untreated or exposed to touch surfaces, was assessed by plaque assay in a RAW 264.7 monocyte macrophage cell line. Copper alloy surfaces were compared to stainless steel as touch surfaces. Results are expressed as plaque forming units (pfu) per cm^2^. The role of Cu(I) or Cu(II) ions and reactive oxygen species (ROS) was assessed using specific chelators and quenchers. Viral RNA was extracted and purified and separated in non-denaturing gel electrophoresis.

## Results

Complete inactivation of approximately 5 x10^4^ pfu per cm^2^ was observed on copper and copper nickel in 5-10 minutes or in 2 hours at room temperature for alloys containing lower percentage copper with an inoculum that dried in seconds, simulating hand contact. Virus exposed to stainless steel retained high infectivity at 2 hours. Inactivation was slower if the virus was inoculated as a wet inoculum simulating vomitus: complete inactivation occurred in 1 hour for copper and copper nickel, with significant reduction on other alloys but not stainless steel. The highest rate of inactivation was observed on immediate contact. These results were similar if virus burden was increased 50-fold. Virus inactivation was faster at 37^o^C and slower at 4^o^C. Cu(II) and particularly Cu(I) ions were essential for loss of infectivity but not superoxide or hydroxyl radicals. Exposure to copper alloys resulted in destruction of the viral genome, preventing potential mutation to copper resistance.

## Conclusion

The results support the use of antimicrobial copper surfaces to reduce the spread of norovirus in high risk areas such as closed environments including health care facilities and cruise ships.

## Disclosure of interest

B. Keevil Grant/Research support from International Copper Association, S. Warnes Grant/Research support from International Copper Association

